# United States Supreme Court

**Published:** 1897-04

**Authors:** 


### United States Supreme Court.

Justice Field, in announcing a decision of this court in a case being tried before that tribunal, said:

"The State, in the exercise of its power to provide for the general welfare of its people, may exact from parties, before they can practice medicine, a degree of skill and learning in that profession upon which the community employing their services may confidently rely; and to ascertain whether they have such qualifications, require them to obtain a certificate or license from a Board or other authority competent to judge in that respect. If the qualifications required are appropriate to the profession, and attainable by reasonable study and application, their validity is not subject to objection because of their stringency or difficulty."

The above decision applies to dentists, and, indeed, to all legally-qualified medical specialists, as well as to the general practioner, and doubtless Justice Field would so construe his decision were his attention called to it.—[Ed. Register.

